# Interaction of Oxygen Molecules with Fe Atom-Doped γ-Graphyne Surfaces: First-Principles Calculations

**DOI:** 10.3390/nano15191479

**Published:** 2025-09-27

**Authors:** Bin Zhao, Jiayi Yin, Zhuoting Xiong, Wentao Yang, Peng Guo, Meng Li, Haoxian Zeng, Jianjun Wang

**Affiliations:** Zhengzhou Key Laboratory of Low-Dimensional Quantum Materials and Devices, College of Physics and Optoelectronic Engineering, Zhongyuan University of Technology, Zhengzhou 450007, China

**Keywords:** γ-graphyne, doped, oxygen activation, dissociation behavior, first-principles calculation

## Abstract

The activation and dissociation of O_2_ molecules play a key role in the oxidation of toxic gas molecules and the oxygen reduction reaction (ORR) in hydrogen–oxygen fuel cells. The interactions between O_2_ molecules and the surfaces of Fe-doped γ-graphyne were systematically explored, mainly adopting the combined method of the density functional theory with dispersion correction (DFT-D3) and the climbing image nudged elastic band (CI-NEB) method. The order of the formation energy values of these defective systems is *E_f_*(*Fe_C_*_2_) < *E_f_*(*Fe_C_*_1_) < *E_f_*(*Fe_D_*_1_) < *E_f_*(*V_C_*_1_) < *E_f_*(*V_D_*_1_) < *E_f_*(*V_C_*_2_) < *E_f_*(*Fe_D_*_2_) < *E_f_*(*V_D_*_2_), which indicates that the process of Fe dopant atoms substituting single-carbon atoms/double-carbon atoms is relatively easier than the formation of vacancy-like defects. The results of ab initio molecular dynamics (AIMD) simulations confirm that the doped systems can maintain structural stability at room temperature conditions. Fe-doped atoms transfer a certain amount of electrons to the adsorbed O_2_ molecules, thereby causing an increase in the O-O bond length of the adsorbed O_2_ molecules. The electrons obtained by the anti-bonding 2π* orbitals of the adsorbed O_2_ molecules are mainly derived from the 3d orbitals of Fe atoms. There is a competitive relationship between the substrate’s carbon atoms and the adsorbed O_2_ molecules for the charges transferred from Fe atoms. In the C1 and C2 systems, O_2_ molecules have a greater advantage in electron accepting ability compared to the substrate’s carbon atoms. The elongation of O-O bonds and the amount of charge transfer exhibit a positive relationship. More electrons are transferred from Fe-3d orbitals to adsorbed O_2_ molecules, occupying the 2π* orbitals of adsorbed O_2_ molecules, further elongating the O-O chemical bond until it breaks. The dissociation process of adsorbed O_2_ molecules on the surfaces of GY-Fe systems (C2 and D2 sites) involves very low energy barriers (0.016 eV for C2 and 0.12 eV for D2). Thus, our studies may provide useful insights for designing catalyst materials for oxidation reactions and the oxygen reduction reaction.

## 1. Introduction

Due to the many excellent properties of graphene materials, more and more researchers are paying attention to new carbon allotropes with a graphene-like structure. As a new two-dimensional material, γ-graphyne is identified as a direct bandgap semiconductor, and it is recognized to have the highest stability among the graphynes family [1,2,3]. In contrast to graphene, graphyne contains two types of hybridized carbon atoms, namely sp^−^ and sp^2−^ hybridized ones, which leads to the unique charge distribution on the surfaces [2,3,4,5,6]. Therefore, surface electronic state modulation can be achieved by doping different types of atoms, thus improving the surface chemical activity and endowing the material surface with peculiar physical and chemical properties [7,8,9,10]. The achievement of producing large-area, high-quality γ-graphyne has been realized recently through mechanochemistry, which has effectively promoted research on the properties and applications of graphyne [11,12]. Some studies indicate that graphynes have promising applications in hydrogen storage, gas sensors and catalysts [13,14,15,16,17,18,19,20,21,22,23]. Similarly to layered graphene, γ-graphyne is considered a potential catalyst for oxidation reactions and the oxygen reduction reaction (ORR). Catalytic activity, meanwhile, is dependent on the adsorption and dissociation of oxygen molecules, which means activated oxygen is vital to these reactions [24,25].

However, the large-area basal plane of γ-graphyne exhibits chemical inertness [26], and free O_2_ molecules do not induce a sensitive response on its (001) surface. This necessitates the search for an effective strategy to activate the γ-graphyne basal plane for catalytic reactions. Theoretical and experimental studies have shown that iron/ruthenium–nitrogen co-doped carbon materials (Fe/Ru-N-C) are considered to be the most active non-precious metal ORR catalysts, with the FeN_4_/RuN_4_ coordination structure being the main catalytic active center [27,28,29]. Some theoretical research results have found that doped graphynes with different configurations have different catalytic performances for the oxygen reduction reaction (ORR). For example, α-graphyne with single B/N doping has low catalytic activity, but singly doped γ-graphyne exhibits certain catalytic activity for ORR [30]. Recently, Cui et al. reported that the anchored transition metal single atoms in nitrogen doped γ-graphyne can achieve efficient CO electrochemical reduction reaction (COER) [31]. Zheng et al. found that the single-atom Ti-doped graphdiyne is beneficial for the dissociation reaction of H_2_O [5]. Our recent work also found that transition metal single-atom-doped γ-graphyne can achieve efficient H_2_O dissociation [26]. Therefore, considering the easy doping characteristics of the graphyne surface, atomic doping can be used to modify the surface of graphyne, achieving a significant improvement in its catalytic performance.

**Figure 1:**
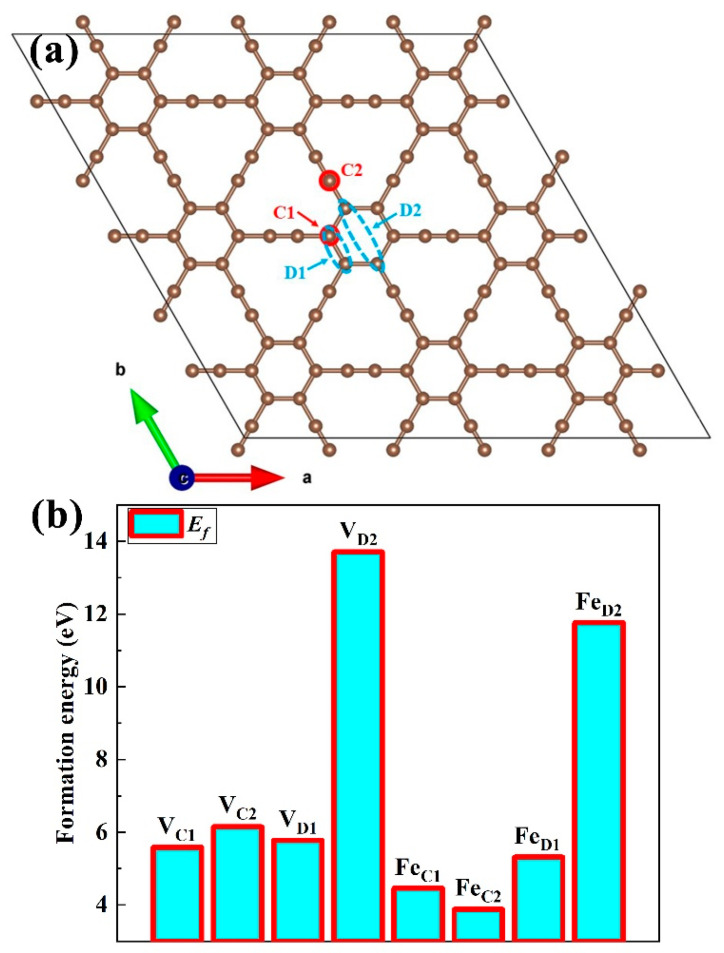
(**a**) Crystal structure of γ-graphyne and four substitution sites for Fe atom, which are labeled as C1, C2, D1 and D2, respectively. (**b**) Formation energies of defective γ-graphyne systems.

In this work, we mainly investigated four doping sites in γ-graphyne: Fe atoms replacing single carbon atoms (C1 and C2 sites in Figure 1) and two adjacent carbon atoms (D1 and D2 sites in Figure 1). The structural stability of these doped systems (GY-Fe system), as well as the activation and dissociation behaviors of O_2_ molecules on the substrates, were studied by using first-principles calculations. The results of ab initio molecular dynamics (AIMD) simulation, binding energies and electronic structural properties show that Fe atoms can be firmly anchored on the surface of γ-graphyne. In particular, O_2_ molecules could be activated effectively on the surfaces of Fe-doped γ-graphyne and further dissociated with a low energy barrier according to the analysis of the optimal dissociation paths. Fe-doped γ-graphyne, as suggested by these results, might be a potential catalyst for oxidation reactions or the oxygen reduction reaction.

## 2. Computational Details

Density functional theory (DFT) was adopted as the theoretical basis for all first-principles calculations, and projector-augmented wave (PAW) pseudopotentials were employed too; these calculations were ultimately performed using the Vienna Ab initio Simulation Package (VASP) [32,33,34,35,36,37]. In this work, the generalized gradient approximation (GGA) served as the approach for addressing exchange–correlation interactions, and within this framework, the Perdew–Burke–Ernzerhof (PBE) functional was employed [38]. Two-dimensional γ-graphyne exhibits hexagonal symmetry, and the space group is P6/mmm. For the 3 × 3 × 1 γ-graphyne super cell (containing 108 C atoms; see Figure 1), geometry optimization and electronic structure calculations employed Gamma-centered 2 × 2 × 1 k-point meshes, with the plane-wave basis set assigned a kinetic energy cutoff of 450 eV. Geometry optimization employed a maximum Hellman–Feynman force of 0.05 eV/Å as its convergence criterion, with electronic iterations set to a convergence value of 1 × 10^−5^ eV. To simulate the adsorption and dissociation behavior of O_2_ molecules on the substrate, a 15 Å thick vacuum layer was introduced to suppress interactions between adjacent layers. To describe the long-range van der Waals (vdW) interactions between the substrate and the adsorbate, the DFT-D3 dispersion-corrected method developed by Grimme was employed [39]. Adsorbed O_2_ dissociation was studied using DFT calculations coupled with the climb image nudged elastic band (CI-NEB) method [40], with charge transfer and redistribution analyzed through Bader charge analysis [41] and the charge density difference (CDD) method.

For describing the interaction between oxygen molecules and the substrate, the adsorption energy of O_2_ molecules designated as *E_ad_*(*O*_2_) is defined by the following expression:

(1) $$\;E_{ad}\left(O_{2}\right)=E\left(sub-O_{2}\right)-E\left(sub\right)-E(O_{2})$$

Here, *E*(*sub-O*_2_) denotes the total energy of the substrate with adsorbed O_2_, while *E*(*sub*) and *E*(*O*_2_) represent the total energies of the bare substrate and the free O_2_ molecule, respectively. The activation energy barrier (*E_a_*) was introduced to describe both the minimum-energy reaction path (MEP) and the transition state (TS) during dissociation, with its definition given as

(2) $$\;E_{a}=E_{TS}-E_{IS}$$

Here, *E*(*TS*) denotes the total energy of the transition state (TS), while *E*(*IS*) represents the total energy of the initial state (IS) in the reaction process, respectively.

## 3. Results and Discussion

### 3.1. Fe Atoms Doped into γ-Graphyne

In general, the formation process of the GY-Fe system should be described as the carbon atom being removed from the pristine γ-graphyne and then the impurity Fe atom being filled in carbon vacancy. As shown in Figure 1a, we selected four substitution sites for Fe atoms, including Fe replacing single carbon atoms of the benzene ring (the C1 site) and acetylene ring (the C2 site), respectively, and Fe substituting double carbon atoms of the benzene ring (D1 and D2 sites). According to some reports [42,43], in order to evaluate the process of impurity Fe replacing the C atom of pristine γ-graphyne, it is essential to calculate the formation energy of the GY-Fe system. The formation energy defined as

(3) $$\;E_{f}=E_{GY-Fe}-E_{GY}-{n_{Fe}\mu }_{Fe}+n_{C}\mu_{c}$$

where $E_{GY-Fe}$ and $E_{GY}$ are the total energies of the GY-Fe system and pristine γ-graphyne. $n_{Fe}$ ($n_{C}$) is the added (removed) number of Fe species (C), and $\mu_{Fe}$ ($\mu_{c}$) is the chemical potential of the Fe (C) atom. $\mu_{Fe}$ is obtained from the Fe bulk material (face-centered cubic phase), and $\mu_{c}$ is obtained from pure γ-graphyne. For example, $\mu_{Fe}=\frac{E_{Fe}(bulk)}{4}=-8.16\;eV$, $\mu_{C}=\frac{E_{GY}}{108}=-8.66\;eV$. As a comparison, we also calculated the formation energy of the carbon vacancy in γ-graphyne (which will be discussed in detail; $E_{f}=E_{GY-Vacancy}-E_{GY}+n_{C}\mu_{c}$). The formation energies of defective γ-graphyne systems are displayed in Figure 1b. According to the results, we find that the values of formation energies are positive, which illustrates that the formation of these defective systems is an endothermic process. The order of the formation energy values of these defective systems is *E_f_*(*Fe_C_*_2_) < *E_f_*(*Fe_C_*_1_) < *E_f_*(*Fe_D_*_1_) < *E_f_*(*V_C_*_1_) < *E_f_*(*V_D_*_1_) < *E_f_*(*V_C_*_2_) < *E_f_*(*Fe_D_*_2_) < *E_f_*(*V_D_*_2_), which indicates that dopant Fe replacing carbon atoms at C2 and C1 sites is relatively easier than the formation of vacancy-like defects in pristine γ-graphyne. Besides that, the formation energy of Fe atoms occupying the D1 site is slightly less than that of the double-carbon vacancy (the D1 site), which indicates that Fe atom occupation at the D1 diatomic site by Fe atoms is relatively easy. However, the calculation results revealed that it is difficult to form D2-type double-carbon vacancy defects on the surface of γ-graphyne. Meanwhile, it is also difficult to form a defective surface with Fe atoms occupying the D2 sites. For comparison, we also investigated the adsorption and dissociation behaviors of oxygen molecules on the surface of GY-Fe (the D2 site).

**Figure 2:**
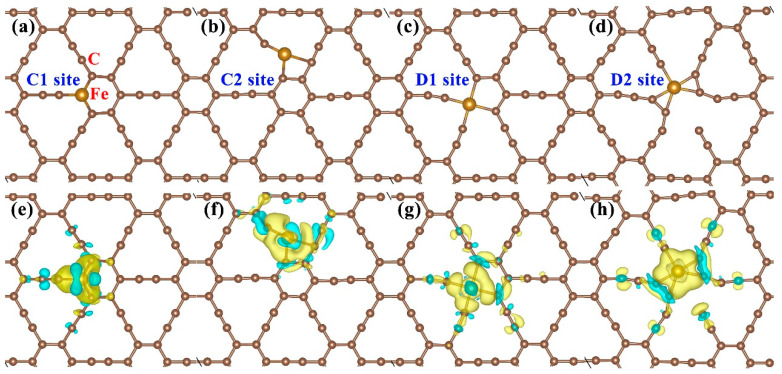
(**a**–**d**) Geometric configurations (top view) and (**e**–**h**) corresponding charge density difference (CDD) of GY-Fe systems. Saddle brown and ochre spheres represent C and Fe atoms, respectively. The yellow (blue) isosurface indicates the charge accumulation (depletion), and the isosurface value is taken to be 0.003 e/bohr^3^.

Before investigating the activation and dissociation behaviors of O_2_ on doped γ-graphyne surfaces with Fe atoms (GY-Fe), we first explored the structural stability and electronic properties of the GY-Fe systems. After structure relaxation, the stable doped substrates were obtained, as shown in Figure 2a–d. It is obvious that impurity Fe atoms could be bound by surrounding carbon atoms. The GY-Fe (C2 and D2 sites) systems face significant structural distortion, which is possibly because the interactions between Fe and neighboring carbon atoms are much stronger relative to the other two doped systems (C1 and D1 sites). The binding energies of these four doped systems are listed in Table 1; they are −6.32 eV, −7.47 eV, −5.66 eV and −7.14 eV for the C1 site, C2 site, D1 site and D2 site, respectively. In view of the distances between Fe and neighboring carbon atoms (*d_Fe-C_*) being less than the sum of the atomic radii of Fe and C atoms, it can be considered that the formation of chemical bonds between Fe and adjacent carbon atoms occurs.

**Figure 3:**
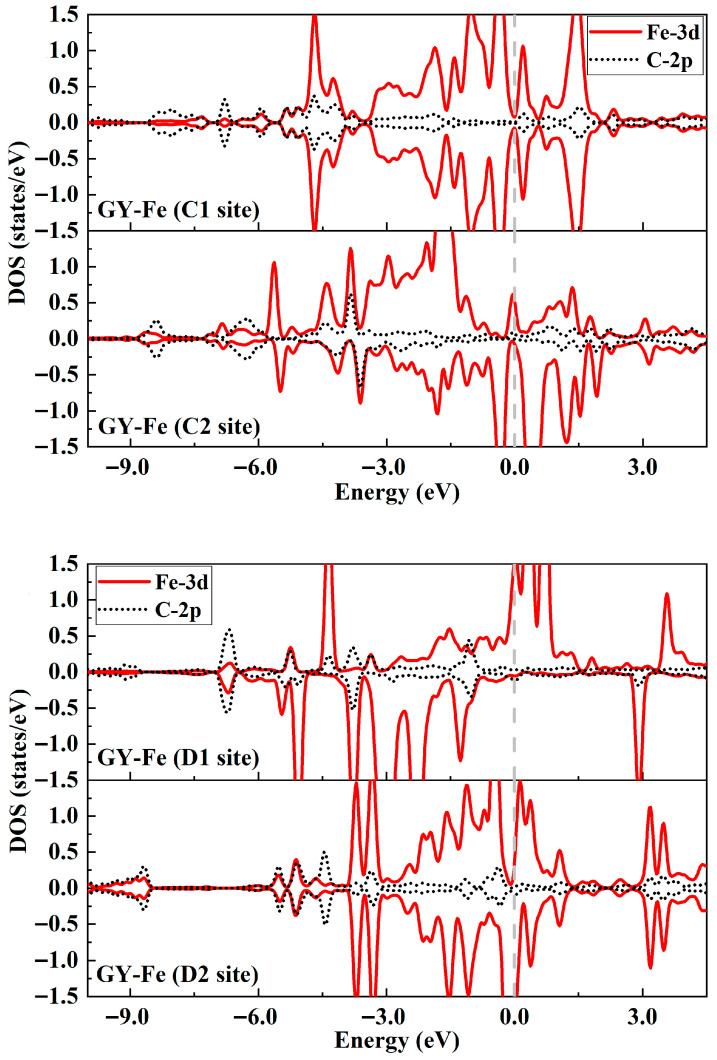
Partial density of states for impurity Fe and neighboring C atoms of GY-Fe systems (C1, C2, D1 and D2 sites). The Fermi level is set to zero.

In order to gain insight into the interactions between Fe and the GY surface, the electronic properties of the four doped systems are discussed in detail. Figure 2e–h display the charge density difference (CDD) of the GY-Fe systems. Charge accumulations occur around the Fe-C chemical bonds, and it can be observed that a certain symmetric distribution of charges exists for the C1 site, D1 site, and D2 site doped systems. Furthermore, Bader charge analysis (Table 1) gives the amount (**∆***Q*) of charges transferring from GY to Fe atoms; they are about ${-0.78\;e}^{-}$, ${-0.82\;e}^{-}$, ${-1.11\;e}^{-}$ and ${-0.85\;e}^{-}$, respectively. Accompanied by charge transfers and charge redistributions, the formation of chemical bonding between Fe and neighboring carbon atoms takes place. In addition, as shown in Figure 3, it is observed that the Fe-3d orbital electronic states are strongly hybridized with the C-2p orbital electronic states, implying a strong interaction between them. Moreover, it is clear that some impurity peaks appear around the Fermi level due to the introduction of Fe into the GY surface, which mainly originates from Fe-3d orbital electronic states, implying that it may be more beneficial for an electron leaping into the conduction band from the valence band.

**Figure 4:**
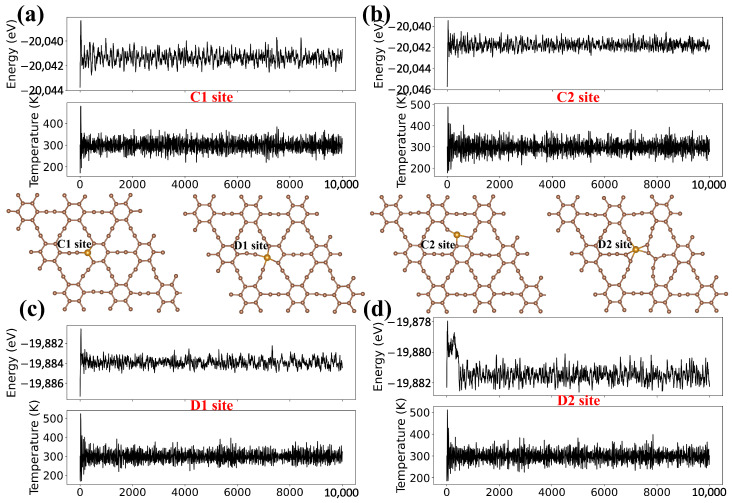
(**a**–**d**) Total energy fluctuation within 10 ps and snapshots of atomic structure of GY-Fe systems (C1 site, C2 site, D1 site and D2 site) during the AIMD simulations at T = 300 K.

Next, the dynamic stability of the GY-Fe systems was evaluated by ab initio molecular dynamics (AIMD) simulations with the NVT ensemble and the Nosé–Hoover thermostat [44], which is implemented in the DS-PAW calculation program under the Device Studio platform [35,45,46]. As shown in Figure 4, snapshots of the atomic structure and the total energy fluctuation within 10 ps of GY-Fe systems (the C1 site, C2 site, D1 site and D2 site) during the AIMD simulations at T = 300 K and 500 K (Figure S1) are presented. It is observed that the geometric structures of GY-Fe systems are well maintained. Moreover, the total energy shows slight oscillations at 300 K, with the oscillation range within ~2 eV. Thus, we think that the structural stability of GY-Fe systems can be maintained at room temperature. In summary, we believe that impurity Fe atoms could be firmly anchored in the γ-graphyne plane based on the analyses of binding energy, electronic properties, and dynamic stability simulations.

### 3.2. Activation and Dissociation Behaviors of O_2_ on GY-Fe Surfaces

**Figure 5:**
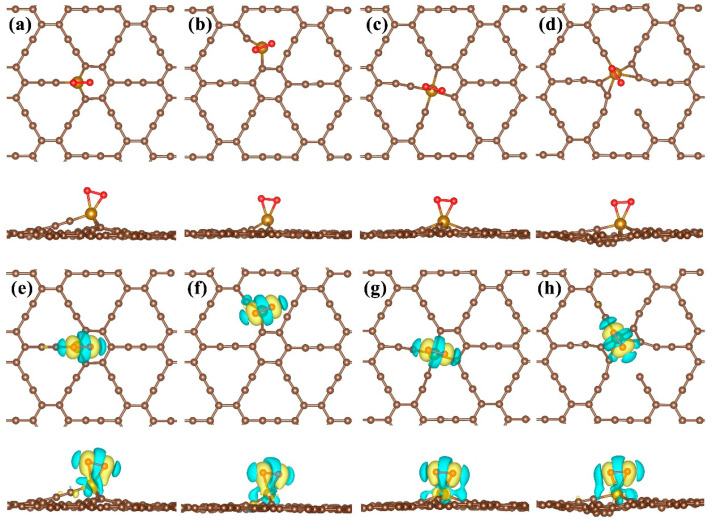
(**a**–**d**) Geometric configurations of and (**e**–**h**) charge density difference in O_2_ molecule adsorption on the GY-Fe surfaces. Saddle brown, ochre and red spheres represent C, Fe and O atoms, respectively. The yellow (blue) isosurface indicates the charge accumulation (depletion), and the isosurface value is taken to be 0.005 e/bohr^3^.

The above calculation results indicate that there could be the formation of stable doped substrates by embedding Fe atoms in the γ-graphyne plane. Thereupon, we further explored the interactions between O_2_ molecules and GY-Fe surfaces. The absolute value of the adsorption energy of O_2_ molecules on the pristine γ-graphyne surface is below 0.007 eV, a result that suggests weak physisorption between O_2_ and the surface. Notably, the adsorption capacity of GY surfaces for O_2_ molecules could be enhanced remarkably when impurity Fe atoms are embedded in the plane. As listed in Table 2, the adsorption energies are −1.73 eV, −2.02 eV, −0.82 eV, and −2.21 eV for the C1 site, C2 site, D1 site, and D2 site, respectively, which implies that there should be chemical adsorption behavior of O_2_ molecules on GY-Fe surfaces. Furthermore, when compared with the results of previous studies [47], it can be observed that the absolute value of the adsorption energy of O_2_ molecules on the GY-Fe surface (C1 and C2 sites) obtained in the current study is relatively smaller. This indicates that the conventional DFT method tends to overestimate the adsorption behavior of O_2_ molecules on the GY-Fe doped surfaces. In Figure 5a–d, the stable adsorption configurations of O_2_ on GY-Fe surfaces are obtained after structural optimization. It can be clearly seen that O_2_ molecules are fixed on the surfaces of GY-Fe systems through the bonding between Fe atoms and two oxygen adatoms. The lengths of Fe-O chemical bonds are about 1.83 Å for these four doped systems. Due to the chemical interactions between adsorbed O_2_ and GY-Fe surfaces, the O-O bonds are nearly parallel to the substrates, and the bond lengths are elongated to 1.38 Å, 1.41 Å, 1.36 Å and 1.36 Å from 1.23 Å (gas phase) at the C1 site, C2 site, D1 site and D2 site, respectively. In fact, the lengths of O-O bonds are very close to the typical value of a superoxide (O_2_^−^) (the bond length is about 1.40 Å), which indicates that O_2_ molecules are activated when they are adsorbed on the GY-Fe surfaces. Activated oxygen usually plays a key role in the oxidation reaction for toxic gas molecules [48] and the oxygen reduction reaction in hydrogen–oxygen fuel cells [24].

**Figure 6:**
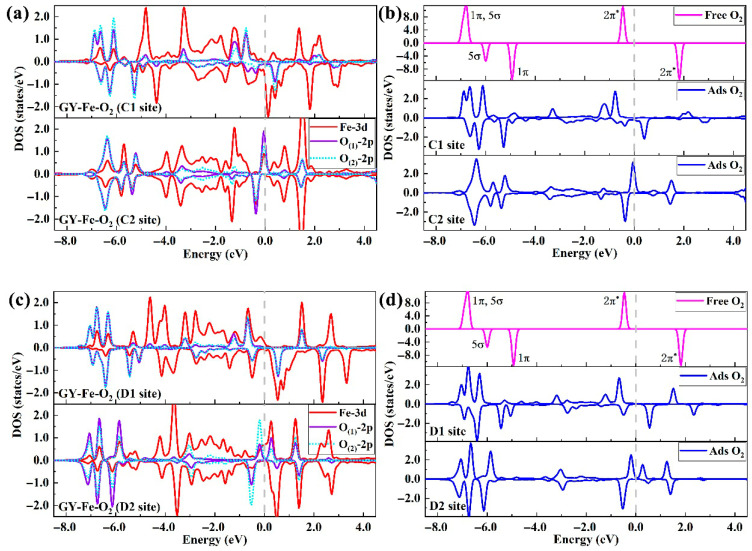
(**a**,**c**) The partial density of states for doped Fe-3d orbitals, as well as O-2p orbitals of adsorbed O_2_ on the GY-Fe surface. The Fermi level is set to 0 eV. (**b**,**d**) The electronic density of states of free and adsorbed oxygen molecules.

To further gain insight into the activation behaviors of O_2_ molecules on GY-Fe surfaces, the spin-polarized partial density of states was calculated. As shown in Figure 6a,c, it is obvious that the electronic states (O_(1)_ and O_(2)_-2p orbitals) of adsorbed O_2_ are sufficiently overlapping with the 3d orbitals of the dopant Fe atom, which directly confirms the formation of Fe-O chemical bonds between GY-Fe surfaces and the adsorbed O_2_ molecules. Furthermore, the localization of electronic states (including 2π*, 1π and 5σ orbitals) is broken and energy levels are broadened in various degrees relative to free O_2_ molecules (Figure 6b,d). Notably, the spin-down 2π* orbital exhibits partial electron occupation, which implies that O_2_ molecules act as electron acceptors during the adsorption process, and then the formation of superoxide anions on GY-Fe surfaces takes place. It can be confirmed from the Bader charge analysis (Δ*Q*), as listed in Table 2, that about ${0.61\;e}^{-}$, ${0.67\;e}^{-}$, ${0.52\;e}^{-}$ and ${0.51\;e}^{-}$ are transferred to the adsorbed O_2_ from the substrates for the C1 site, C2 site, D1 site and D2 site, respectively. Besides that, charge redistributions occurred between adsorbed O_2_ and Fe atoms because of the charges transferring between adsorbed O_2_ and substrates. The results of the charge density difference (CDD) are shown in Figure 5e–h; charges accumulated around Fe-O chemical bonds, and charge depletion happened between the two O adatoms, which can serve as evidence that O_2_ molecules are activated on GY-Fe surfaces. Moreover, according to the adsorption parameters of O_2_ molecules on different doped surfaces (Table 2), the elongated lengths of O-O bonds and the amount of charge transfer exhibit a positive relationship. That is, the greater the amount of charge transferred to the adsorbed O_2_, the larger the elongation of the O-O bond length. To provide a reasonable explanation, it is necessary to discuss the changes in the density of states of O_2_ molecules on GY-Fe surfaces before and after adsorption. For example, as shown in Figure 6b (C2 site), it is obvious that the spin-down 2π* orbital is partially filled with electrons (the peak of occupied states is located at −0.37 eV), but this is an unoccupied orbital for free O_2_ molecules (the peak of unoccupied states is located at 1.83 eV). It is also because a certain amount of electrons were injected into the anti-bonding 2π* orbital of adsorbed O_2_ that the O-O bond length was significantly elongated. Similar features of electronic states for the other three doped systems (C1, D1 and D2 sites) also appeared. On the whole, the chemical inertness of the surface can be broken when impurity Fe atoms are doped in the γ-graphyne plane, and O_2_ molecules are activated through charge transfer and charge redistribution between them and the substrates. However, there is still a certain amount of Fe-3d orbital electrons that need to be transferred to the carbon atoms in the substrates, and the amount of electron transfer is related to the Fe-C coordination structure (Table S1). In the C1 and C2 systems, O_2_ molecules have a greater advantage in electron accepting ability compared to the substrate’s carbon atoms, which means that the activation of O_2_ molecules on doped surfaces is related to their surface structure.

**Figure 7:**
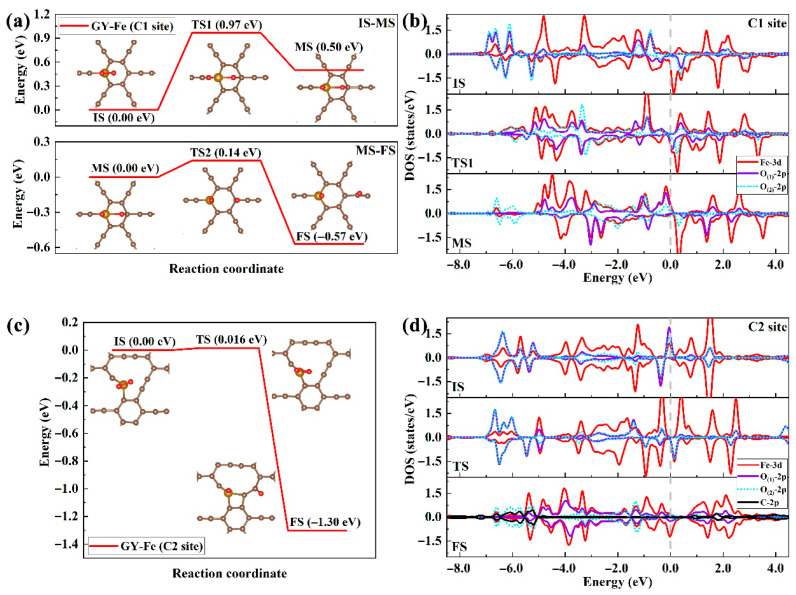
(**a**,**c**) The dissociation paths of adsorbed O_2_ on GY-Fe surfaces (C1 and C2 sites), including the initial state (IS), transition state (TS), and final state (FS) configurations along the minimum-energy path (MEP). (**b**,**d**) The density of electronic states during the dissociation process. Saddle brown, ochre and red spheres represent C, Fe and O atoms, respectively.

**Figure 8:**
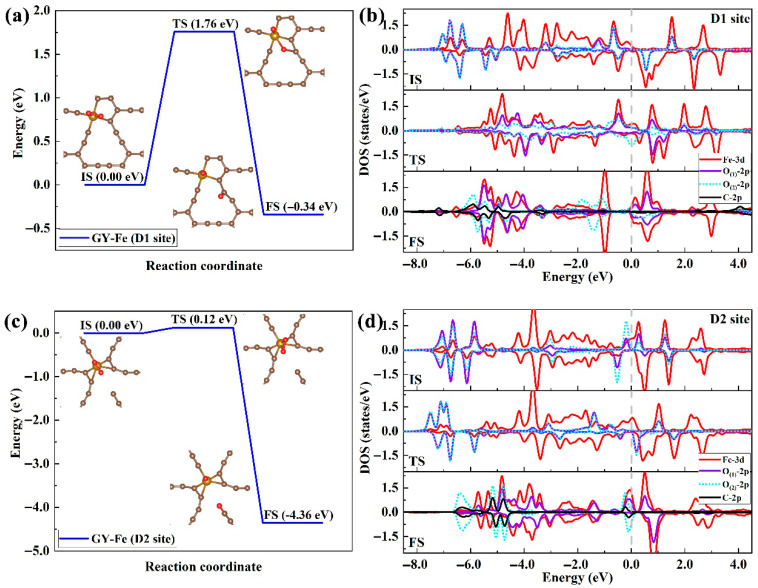
(**a**,**c**) The dissociation paths of adsorbed O_2_ on GY-Fe surfaces (D1 and D2 sites), including the initial state (IS), transition state (TS), and final state (FS) configurations along the minimum-energy path (MEP). (**b**,**d**) The density of electronic states during the dissociation process. Saddle brown, ochre and red spheres represent C, Fe and O atoms, respectively.

Based on the above studies, the dissociation behaviors of adsorbed O_2_ molecules on GY-Fe surfaces were further explored by the climbing image nudged elastic band (CI-NEB) method. As shown in Figure 7 and Figure 8, the schematics of the minimum-energy reaction paths (MEPs) for O_2_ dissociation on GY-Fe surfaces are described, including the dissociation energy barriers, as well as the configurations of the initial state (IS), transition state (TS) and final state (FS) in the reaction process. For the C1 site (Figure 7a IS-MS), adsorbed O_2_ could be dissociated into two adsorbed O atoms with an energy barrier of about 0.97 eV: one O is located at the top site of the Fe atom, and the other O adatom moves to the vicinity of the para-position carbon atom. More electrons are transferred from Fe-3d orbitals to adsorbed oxygen molecules, occupying the 2π* orbitals of adsorbed O_2_ molecules, further elongating the O-O chemical bond until the bond gradually breaks. As shown in Figure 7b, it is very clear to observe that the density of state peak of the previously unoccupied spin-down O-2p (O_(1)_-2p and O_(2)_-2p) orbitals shifts towards the Fermi level and gradually becomes occupied. By comparing the total energy of the initial state (IS) with that of the middle state (MS), it can be concluded that the dissociation process is an endothermic reaction that absorbs about 0.50 eV of energy. Although the O-O bond has been broken at the saddle point, the two O adatoms remain bonded with the Fe atom in the dissociation process. As shown in Figure 7a (MS-FS), the adsorbed O atom located above the carbon atoms of the benzene ring can further migrate to the top site of the carbon atoms in the acetylene ring. During this process, only an energy barrier of 0.14 eV needs to be overcome, and a reaction heat of 0.57 eV is released. For the entire dissociation process (IS-MS-FS), the total energy change is calculated to be −0.07 eV, which indicates that the dissociation reaction of O_2_ molecules on the surface of GY-Fe (the C1 site) can proceed (or is thermodynamically feasible).

As shown in Figure 7c, the O-O bond of adsorbed O_2_ at the C2 site can be broken by overcoming an energy barrier of about 0.016 eV, and a more stable dissociation configuration (FS) is obtained through the release of about 1.30 eV. Analyzing the structure of FS, adsorbed O_2_ on the GY-Fe surface (C2 site) completely dissociates, and one of the O atoms migrates to the vicinity of a carbon atom in the acetylene ring. Moreover, the reconstruction of chemical bonds between the impurity Fe and carbon atoms of the acetylene ring can also be observed. In addition, as explained regarding the relationship between the elongated lengths of O-O bonds and the amount of charge transfer in the previous section, we believe that the length of the O-O bond can be further elongated if more charges are transferred to the activated O_2_. As shown in Figure 7d, it is clearly seen that there is a hybridization peak around at the Fermi level in the DOS of the C2 site, and the hybridization of electronic states (partially occupied spin-up electronic states) mainly originates from O-2p (O_(1)_-2p and O_(2)_-2p) orbitals (which are the main components of the 2π* orbital (in Figure 6b C2 site)) of adsorbed O_2_ and the Fe-3d orbital. The distribution of such electronic states is more favorable for the transfer of electrons from the GY-Fe substrate to the adsorbed O_2_ molecules during the dissociation process. Subsequently, it can be observed that in the transition state, the peak of the electron density of states of the originally semi-occupied spin-up 2π* orbitals gradually shifts beyond the Fermi level towards the valence band and is occupied by electrons. On the basis of these results, compared with the case of the C1 site, the dissociation reactions at the C2 site should be able to proceed spontaneously because of the lower energy barrier and greater amount of thermal energy released.

Similarly, we also simulated the dissociation process of O_2_ molecules on the GY-Fe (D1 and D2 sites) systems. For the D1 site (Figure 8a), the energy barrier for adsorbed O_2_ that separates into two O adatoms is about of 1.76 eV, and the total energy of system decreases by 0.34 eV after dissociation. The dissociation energy barrier is much higher than that of the above two systems, which indicates that the dissociation of O_2_ molecules on the GY-Fe surface (at the D1 site) requires a large amount of external energy, although the dissociation process is an exothermic reaction. By analyzing the changes in the electronic density of states (DOSs) during the dissociation process (Figure 8b), we find that this process also involves charge transfer and the occupation of spin-down anti-bonding 2π* orbitals and ultimately achieves the cleavage of the O-O chemical bond. However, the energy level difference between the hybridized overlapping peaks (resulting from the hybridization of spin-down O-2p and Fe-3d orbitals) located on both sides of the Fermi level is relatively large, which may be unfavorable for the charge transfer from Fe atoms to adsorbed O_2_ molecules. In addition, in the GY-Fe system (the D1 site), the amount of charge transferred from Fe atoms to the substrate carbon atoms is higher than that in the other three systems (about ${1.11\;e}^{-}$). However, when oxygen molecules are adsorbed on its surface, the amount of charge transferred from the substrate to the oxygen molecules is the lowest (about $0.52\;e^{-}$). Therefore, it can be inferred that there is competition for charge transfer between the carbon atoms and the adsorbed O_2_ molecules, which results in the lowest absolute value of the adsorption energy on its surface and the highest energy barrier during the dissociation reaction.

Different to the cases of the above three doped systems, for the D2 doped site (Figure 8c), the dissociation reaction of adsorbed O_2_ molecules requires an extremely low energy barrier (0.12 eV), and the total energy decreases sharply by about 4.36 eV from the initial state (IS) to the final state (FS). The results illustrate that the dissociation of O_2_ molecules on the GY-Fe (D2 site) surface can proceed continuously with the help of a large amount of released reaction heat. As shown in Figure 8d, it can be clearly seen that unoccupied electronic states of spin-up O_(1)_-2p and O_(2)_-2p orbitals (that is, the 2π* orbital of adsorbed O_2_ molecules in Figure 6c,d) and unoccupied states of the Fe-3d orbital completely overlap at the same level position (~0.27 eV). In fact, the electronic states of the Fe-3d orbital close to the Fermi level are partially occupied, and this energy region crosses the Fermi level, which indicates that more electrons can be further transferred into the unoccupied electronic states of the spin-up 2π* orbital. Indeed, from the electronic density of state (DOS) results obtained in the dissociation process, the originally unoccupied 2p orbital DOS peaks were observed to gradually shift towards the Fermi level and become occupied. Therefore, based on the above results, we can conclude that the dissociation of O_2_ molecules on the GY-Fe (D2 site) surface can be achieved with only a small amount of external energy.

## 4. Conclusions

In summary, the activation and dissociation behaviors of O_2_ molecules on GY-Fe surfaces were systematically explored by using the first-principles calculations coupled with the CI-NEB method. The formation energy results show that the replacement of carbon atoms by Fe dopants (at C1, C2 and D1 sites) occurs relatively more easily than the formation of vacancy-like defects in γ-graphyne. From the results of electronic properties, the formation of chemical bonds between Fe atoms and adjacent carbon atoms indicates that Fe atoms are bound to the γ-graphyne surface. The results of ab initio molecular dynamics (AIMD) simulation confirmed that the GY-Fe systems (with C1, C2, D1, and D2 doped sites) can maintain structural stability at room temperature (300 K). O_2_ molecules exhibit chemical adsorption behavior on the surfaces of all four doped substrates, while different doped surfaces show varying degrees of activation toward them. The results of electronic structure properties show that Fe-doped atoms transfer a certain amount of electrons to the adsorbed O_2_ molecules, thereby causing an increase in the O-O bond length of the adsorbed O_2_ molecules. More electrons are transferred from Fe-3d orbitals to adsorbed oxygen molecules, occupying the 2π* orbitals of adsorbed O_2_ molecules, further elongating the O-O chemical bond until the bond gradually breaks. Adsorbed O_2_ molecules on the GY-Fe surfaces can be dissociated into two O adatoms with a lower energy barrier at the C1, C2, and D2 doped sites; the corresponding low dissociation energy barriers are about 0.97 eV, 0.016 eV, and 0.12 eV, respectively. Especially, these three dissociations are all exothermic reactions with the release of about 0.07 eV, 1.30 eV, and 4.36 eV, respectively. Therefore, the surfaces of γ-graphyne doped with Fe atoms are beneficial for the activation and dissociation of O_2_ molecules, and this may serve as a feasible strategy for designing catalyst materials for oxidation reactions and oxygen reduction reactions.

## Figures and Tables

**Table 1:** Calculated results for Fe-embedded graphyne systems, including the distance *d_Fe-C_* between Fe and neighboring carbon atoms, the binding energies of Fe atoms on graphyne (*E_b_*), the charge transfer from graphyne to Fe atom (∆*Q*).

Position	*d_Fe-C_* (Å)	*E_b_* (eV)	∆*Q* (*e*^−^)
**C1**	1.80/1.75/1.80	−6.32	−0.78
**C2**	1.66/1.86/2.17	−7.47	−0.82
**D1**	1.84/1.84/1.98/1.98	−5.66	−1.11
**D2**	1.80/1.89/1.89/1.80/1.96	−7.14	−0.85

**Table 2:** Adsorption parameters of O_2_ molecules on GY-Fe surfaces, including adsorption energy (*E_a_*); the length of Fe-O chemical bond (*L_Fe-O_*), the bond length of adsorbed O_2_ molecules (*L_O-O_*), charge transfer from the substrate to adsorbed O_2_ molecules (∆Q).

Position	*E_a_* (eV)	*L_Fe-O_* (Å)	*L_O-O_* (Å)	Δ*Q* (*e*^−^)
**C_1_**	−1.73	1.90/1.83	1.38	0.61
**C_2_**	−2.02	1.83/1.83	1.41	0.67
**D_1_**	−0.82	1.86/1.86	1.36	0.52
**D_2_**	−2.21	1.78/1.99	1.36	0.51

## Data Availability

All data are included in the article and the Supplementary Materials.

## Supplementary Materials

The following supporting information can be downloaded at: https://www.mdpi.com/article/10.3390/nano15191479/s1, Figure S1: (a–d) Total energy fluctuation within 10 ps and snapshots of atomic structure of GY-Fe systems (C1 site, C2 site, D1 site and D2 site) during the AIMD simulations at T = 500 K. Table S1: Charge transfer amounts between C-substrates, adsorption O_2_ molecules and Fe atoms, as well as the bond lengths of adsorbed O_2_ molecules (L_O-O_).
