# Supplementary material for: Interaction of Oxygen Molecules with Fe Atom-Doped γ-Graphyne Surfaces: First-Principles Calculations
Source: Nanomaterials (Basel). 2025 Sep 27;15(19):1479. doi: 10.3390/nano15191479 (PMC12526025; doi:10.3390/nano15191479)
Supplement: Supplementary file 1 [file nanomaterials-15-01479-s001.zip › nanomaterials-3871363-supplementary.pdf]

Supporting Information

Interaction of Oxygen Molecules with Fe Atom-Doped γ-Graphyne Surfaces: First-Principles Calculations

Bin Zhao *, Jiayi Yin, Zhuoting Xiong, Wentao Yang, Peng Guo and Meng Li, Haoxian Zeng and Jianjun Wang *

College of Physics and Optoelectronic Engineering, Zhengzhou Key Laboratory of Low-Dimensional Quantum Materials and Devices, Zhongyuan University of Technology, Zhengzhou 450007, China

* Correspondence: zhaobin@whu.edu.cn (B.Z.); jjwang@zut.edu.cn (J.W.)

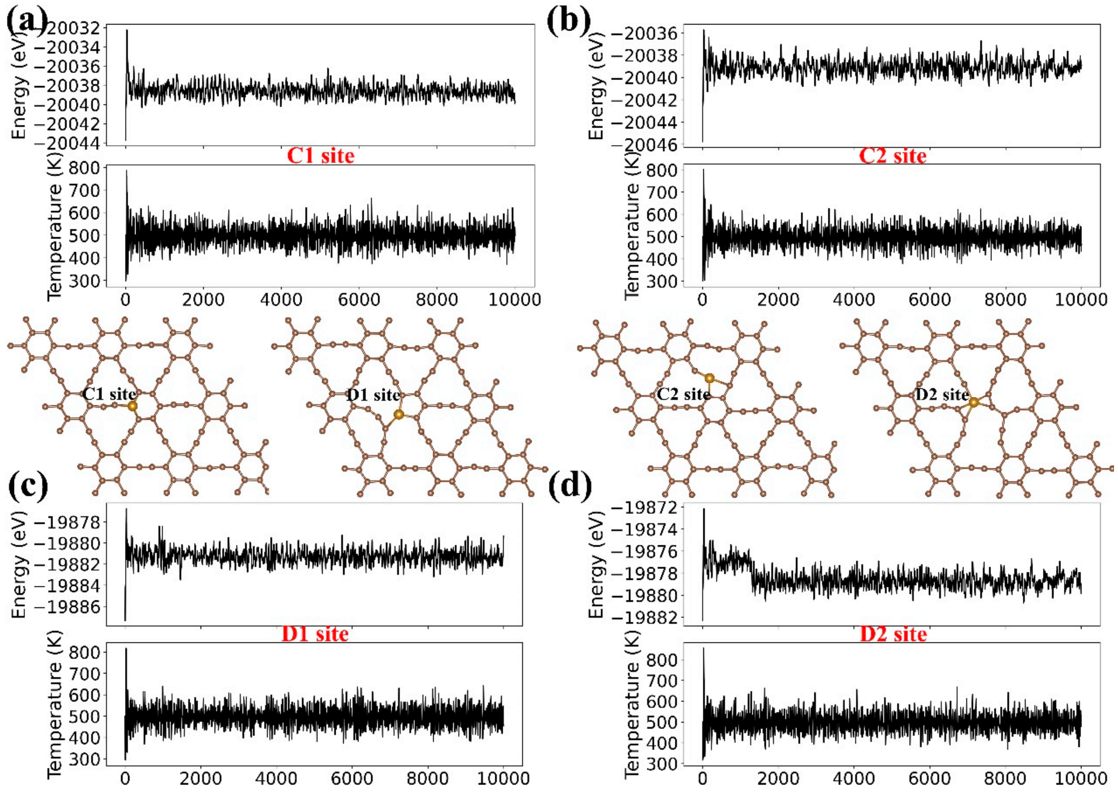

Figure S1 (a)-(d) Total energy fluctuation within 10 ps and snapshots of atomic structure of GY-Fe systems (C1 site, C2 site, D1 site and D2 site) during the AIMD simulations at T = 500 K.

Table S1 Charge transfer amounts between C-substrates, adsorption O_2 molecules and Fe atoms, as well as the bond lengths of adsorbed O_2 molecules ($L_{O\text{-}O}$).

Type	Charge transfer amount (ΔQ (e^{-}))			$L_{O\text{-}O}$(Å)
	Fe	C-substrate	Ads-O_2	
**C1**	1.091309	0.477864	0.613445	1.38325
**C2**	1.03246	0.364917	0.667543	1.40498
**D1**	1.163406	0.647125	0.516281	1.36294
**D2**	1.033812	0.523246	0.510566	1.35562
